# Multiorgan ECV as measured by EQ-MRI in systemic amyloidosis

**DOI:** 10.1186/1532-429X-15-S1-O34

**Published:** 2013-01-30

**Authors:** Sanjay M Banypersad, Steve Bandula, Daniel Sado, Jennifer H Pinney, Simon D Gibbs, Viviana Maestrini, Marianna Fontana, Steven K White, Shonit Punwani, Stuart Taylor, Philip N Hawkins, James Moon

**Affiliations:** 1 MRI, The Heart Hospital, London, UK; 2 National Amyloidosis Centre, Royal Free Hospital, London, UK; 3 Radiology, University College Hospital, London, UK

## Background

Systemic AL Amyloidosis causes multiorgan dysfunction through interstitial expansion. We measured the extracellular volume fraction (ECV) in the : heart, liver, spleen and skeletal muscle in healthy volunteers and patients with Systemic Amyloidosis to test the hypotheses that (1) tissue ECV is greater in systemic AL Amyloidosis than in health and (2) ECV tracks organ amyloid burden.

## Methods

Healthy volunteers (n=70; 35 male; 35 female; median age 46 years) and patients with systemic AL Amyloidosis (n=56; 36 male; 20 female; median age 62 years), with clinical indications for CMR scanning, additionally underwent multi-organ ECV measurement. Technical details were: gadoteric acid, 0.1mmol/Kg plus infusion, multibreath-hold T1 measurement and equilibrium imaging of heart, liver spleen and biceps muscle at 1.5T (Siemens Avanto). Amyloidosis patients also underwent serum amyloid P component (SAP) scintigraphy to score liver and spleen involvement by amyloid.

## Results

ECV of the heart, liver, spleen and muscle was significantly elevated in patients with amyloidosis (0.40, 0.33, 0.42 and 0.12 respectively) compared to healthy controls (0.25, 0.30, 0.34 and 0.09 respectively) (P<0.001). ECV measured in the liver and spleen tracked increasing organ amyloid burden assessed by SAP scintigraphy (P<0.001). In healthy volunteers, ECV varied between different organs, being highest in the spleen and lowest in skeletal muscle.

## Conclusions

The cardiac ECV technique measures cardiac amyloid burden, but can be translated into also in other tissues and organs in the body. Here its use is validated against the gold standard of SAP scanning in the liver and spleen. ECV measurement may represent a key technique for measuring ECV increase in systemic diseases.

## Funding

We receive money from Glaxo Smith Kline for some of our studies.

**Figure 1:**
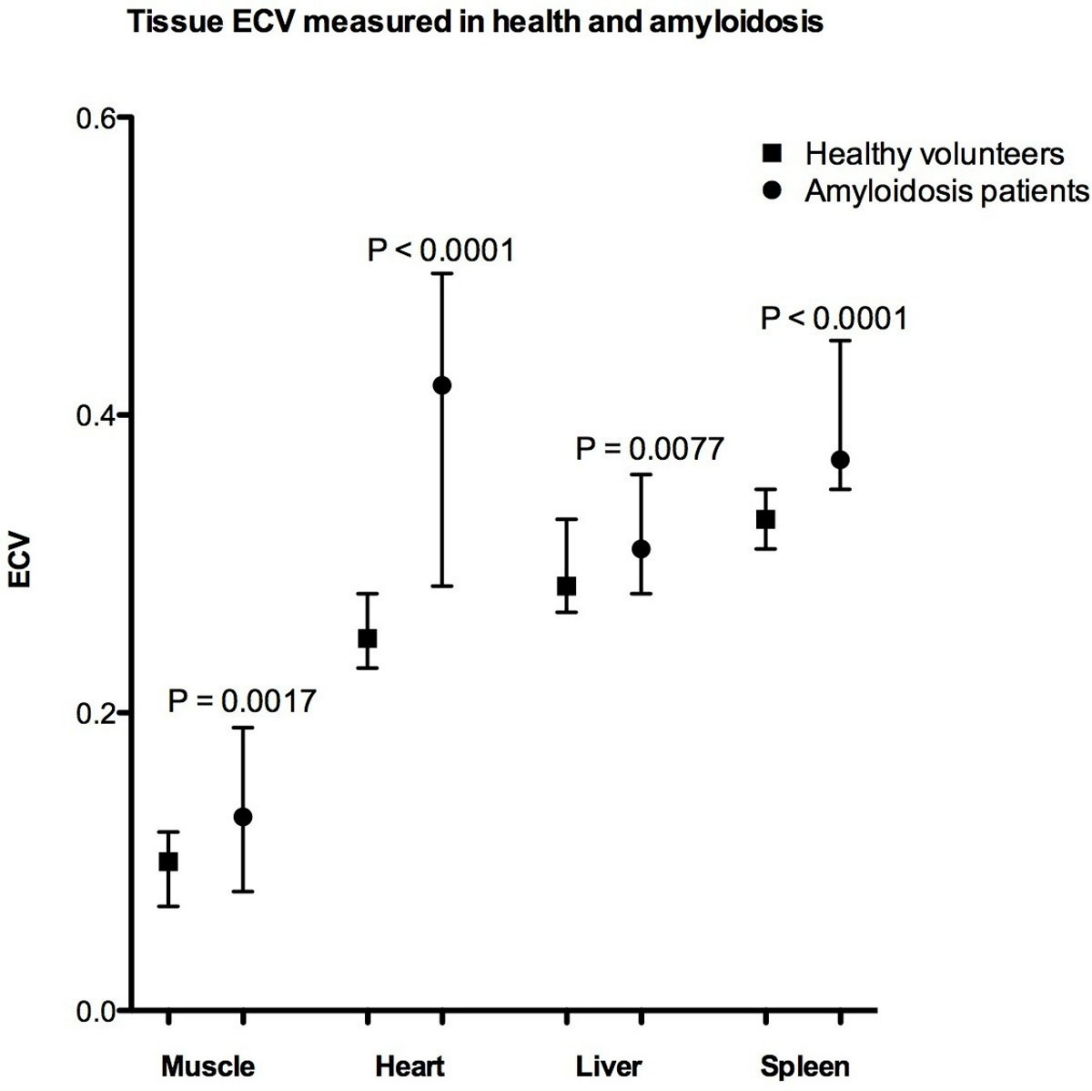
comparing mean ECVs of heart, liver, spleen and biceps muscle in normal volunteers VS patients with amyloidosis.

**Figure 2:**
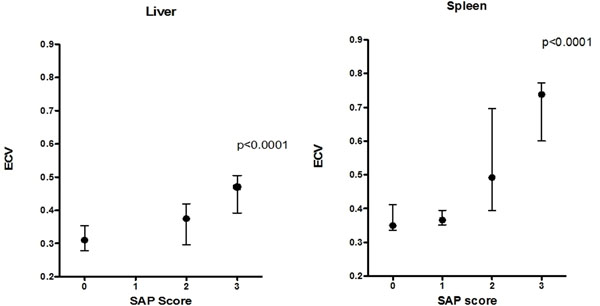
showing ECV tracking amyloid burden when compared to SAP imaging score for liver (left) and spleen (right).

